# A Comparison between Cytotoxicity Induced by Two Resin Based Sealers (2Seal and AH Plus) in Saos-2 and MG-63 Cell Lines

**Published:** 2012

**Authors:** Maryam Ehsani, Ebrahim Zabihi, Hamed Gharouee

**Affiliations:** 1 *Dental Materials Research Center, School of Dentistry, Babol University of Medical Sciences, Babol, Iran.*; 2 *Department of Endodontics, School of Dentistry, Babol University of Medical Sciences, Babol, Iran.*; 3 *Cellular and Molecular Biology Research Center, Babol University of Medical Sciences, Babol, Iran.*; 4 *Department of Pharmacology & Physiology, School of Medicine, Babol University of Medical Sciences, Babol, Iran.*

**Keywords:** Cytotoxicity, AH Plus, 2Seal, osteosarcoma, Saos 2, MG-63

## Abstract

The aim of this study was to evaluate and compare the cytotoxicity induced by two resin-based sealers, 2Seal and AH Plus, in two osteoblast-like cell lines, MG-63 and Saos-2. Using sterile discs of both sealers in complete media, 24- and 72-h extracts were prepared. The extracts were exchanged with Saos-2 or MG-63 cell culture media at 75% confluence, and after 24 h incubation, cell viability tests were performed for each extract and cell line using MTT and trypan blue dye exclusion assays. Corresponding incubated media were used as negative control groups. For both extracts and sealers, cytotoxicity was observed in both cell lines. For Saos-2, there was no statistical difference in toxicity between the sealers for either extract (p > 0.05). For MG-63, the 2Seal 24-h extract and the AH Plus 72-h extract had greater cytotoxicity than the other extracts (p < 0.05(. Both AH Plus and 2Seal demonstrated significant cytotoxicity in these two cell lines. In contrast to 2Seal, the cytotoxicity of AH Plus in the MG-63 cell line increased with extraction time from 24 to 72 h. The AH Plus and 2Seal 24-h extracts showed different levels of cytotoxicity in the MG-63 cell line, while in the Saos-2 cell line there were no detectable differences. This may reflect higher sensitivity of the MG-63 cell line compared to Saos-2 toward cytotoxicity induced by these two sealers, or different kinetics of toxicant release from the sealers.

Substances used for root canal sealing along with endodontics treatment procedures have improved considerably during the last two centuries (1). Despite of great achievements in this field, investigations are ongoing toward developing materials with better physico-chemical properties and lower toxicities. The ideal root canal sealer should prevent penetration of periapical exudates into root canal, prevent recurrence of infection, and provide a microenvironment suitable for tissue healing (2, 3). On the other hand, biocompatibility of the root canal sealers, which could be directly or indirectly in contact with periapical tissues, is very important (4-6) and could affect the healing processes (7). Cell culture based cytotoxicity assays for medical devices and dental materials have got great approvals in the recent decades compared to exhaustive and time consuming in vivo models (4, 7). 

Numerous cell lines including those obtained from human periodontal fibroblasts have been used for dental materials cytotoxicity assays(8, 9). Also cell lines originated from tissues other than periapical or human oral cavity (e.g. 3T3, Hela, V79…) (7, 10, 11) have been used in these assays. However, in order to have a better prediction on biocompatibility of tested compound, it is preferred to use cell lines with similar characteristics and phenotypes to dental and periapical tissues (12, 13). Since osteoblasts play an important role in healing dental and apical tissues, we chose two osteosarcoma cell lines with human origin “Saos-2 and MG-63” (14-16). 

In view of the fact that the chemical composition of different sealing materials varies from one type to another, the *in vitro* biocompatibilitiy results would depend on the selected method of cytotoxicity assay (6). In addition to duration of extraction, the type of cell line, and exposure method would also affect the *in vitro* biocompatibility results. Some materials do not release toxic substances so much but show deleterious effects when come to contact with cells or tissues. At these cases, putting set discs directly in the culture vessels would simulate the *in vivo* condition and more likely detect cytotoxic effects. Gutta-percha is one of the most common used root canal filling materials so far which has a very good biocompatibility (7) but other sealing materials such as zinc oxide-eugenol cement, calcium hydroxide-based, and resin-based sealers release toxic substances and show different degrees of cytotoxicities (5, 9). AH Plus as a well known epoxy resin-based sealer, has shown good properties for successful endodontic therapy including less formaldehyde release, hence lower cytotoxicity in many cell lines (8, 17, 18). As a relatively new introduced sealing material, 2Seal has many physico-chemical properties in common with AH Plus, including its epoxy-amine resin based composition and according to the manufacturer it does have minimal toxicity on living tissues (19). In this study we aimed to evaluate the cytotoxicity induced by 2Seal and compare it with its common used congener AH Plus on two osteosarcoma (fibroblast like) cell lines, Saos-2 and MG-63.

## Methods and Materials

**Cell culture condition**

Saos-2 and MG-63 cell lines (Pasteur Institute, Iran) were seeded in 24 well plates (1.5×10^5^ cells/well) with RPMI-1640 (Sigma-Aldrich, UK) supplemented with 10% FBS (Invitrogen, UK) and 1% PenStrep® (Sigma-Aldrich, UK). After 24 hours, when cells reached 70-80% confluence, the media were changed by the corresponding sealers extracts and after another 24 hours the viability of the cells were measured. 

**Preparation and extraction of the sealers**

According to the manufacturers’ instruction, components of either AH Plus (DENTSPLY DETREY GmbH, Germany) or 2Seal (Roydent Dental Products, USA) were mixed and cylindrical discs (3 mm diameter and 3 mm thickness) were made in a laminar flow safety cabinet using a stainless steel mould. Then discs were aseptically incubated at 37^o^C for 24 hrs to be completely set. Each disc was drawn in 1 ml complete media (RPMI-1640+10% FBS+1% PenStrep®) in 24 wells cell culture plates. The supernatants were collected after 24 or 72 hrs and replaced with either MG-63 or Saos-2 culture media. Complete media incubated in empty wells was used as negative control.

**Sealers cytotoxicity assays**

All experiments were performed in triplicates. The viability of the cells was measured after Saos-2 or MG-63 cell lines exposure to the sealers extracts using MTT assay (20) and Trypan blue dye exclusion (TB) method (21). Briefly, after washing the cells with D-PBSA, 200µl of MTT (Sigma-Aldrich, UK) solution in PBS (5 mg/ml) was added to each well and incubated for 4 hrs at 37^O^C. The purple/blue formazan precipitate was dissolved in 800 μl of acidic isopropyl alcohol (0.04 N) and the colored solution absorbance wasread at 570 nm and 630 nm (as reference wavelength) using a UV-Vis spectrophotometer. For TB assay, numbers of viable cells after 24 hours exposure to the extracts were counted for each triplicate and presented as percentage of the control.

**Statistical Analysis**

The results have been presented as Mean ± Standard Error. Different groups’ means were compared by t-Student and one-way analysis of variance (ANOVA) and Tukey's Post Hoc tests. The statistical significance was set at p< 0.05.

**Fig 1:**
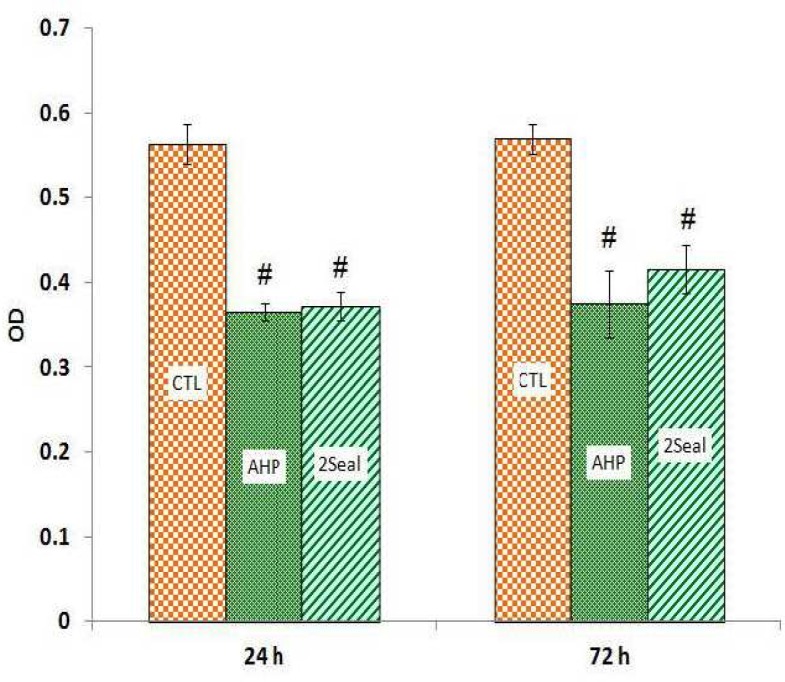
AH Plus (AHP) and 2Seal cytotoxicity on Saos-2 cell line with two differnt extraction times using MTT assay. # significant difference with control group (CTL). * significant difference with other test group

**Fig 2:**
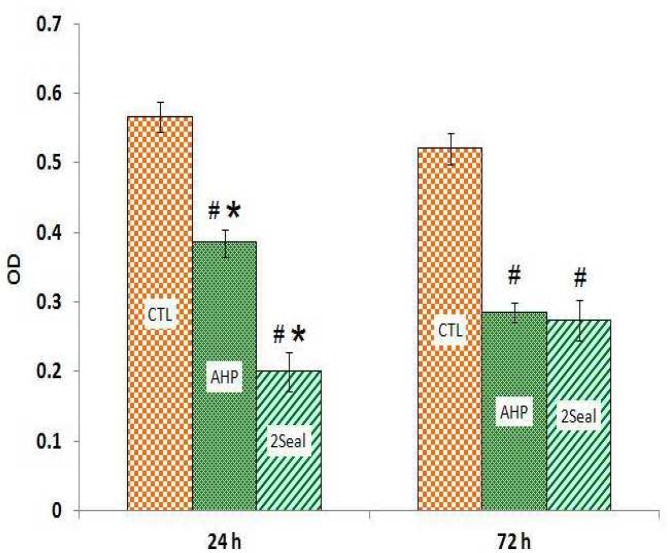
AH Plus (AHP) and 2Seal cytotoxicity on Saos-2 cell line with two differnt extraction times using MTT assay. # significant difference with control group (CTL). * significant difference with other test group

**Fig 3:**
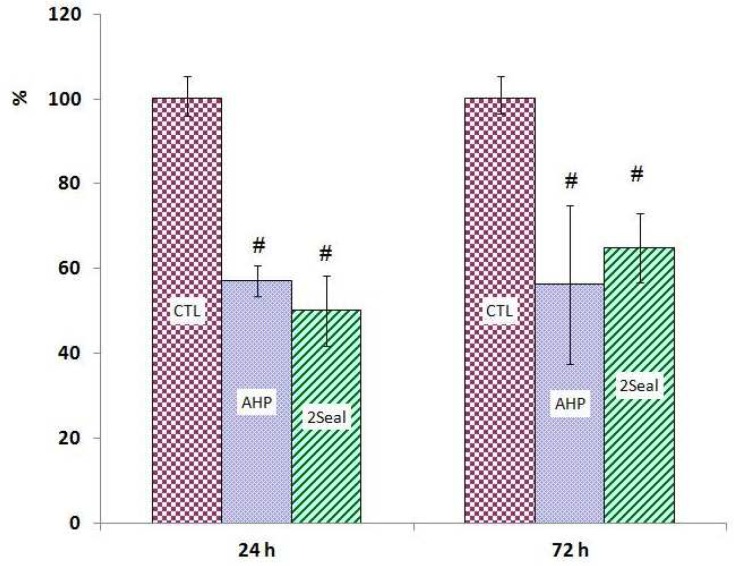
AH Plus (AHP) and 2Seal cytotoxicity on Saos-2 cell line with two different extraction times using trypan-blue exclusion technique. # significant difference with control group (CTL). * significant difference with other test group

**Fig 4:**
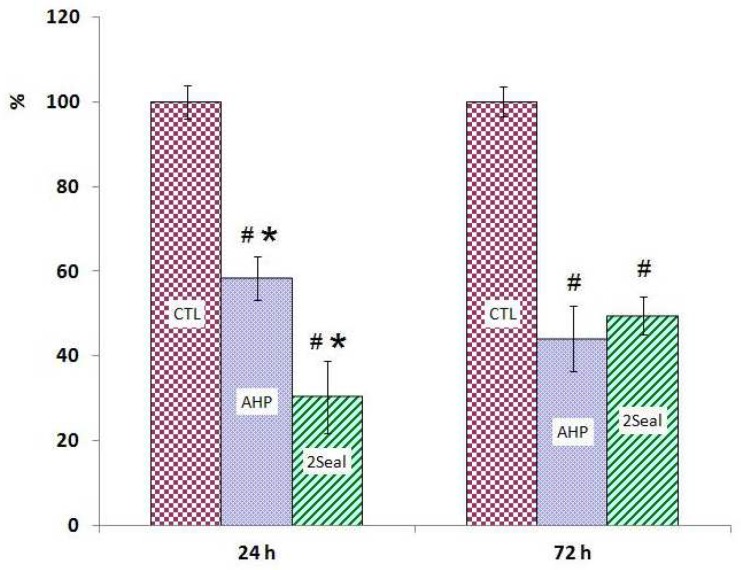
AH Plus (AHP) and 2Seal cytotoxicity on MG-63 cell line with two different extraction times using trypan-blue exclusion technique. # significant difference with control group (CTL).* significant difference with other test group

The results of MTT assays for both 24 and 72 hrs extracts show significant cytotoxic effects in MG-63 and Saos-2 cell lines induced by 2Seal and AH Plus (Fig. 1 and 2). There were no statistically differences between 2Seal and AH Plus cytotoxicities in Saos-2 cell line with both 24 and 72 hrs extracts (Fig. 1). However, there was a higher cytotoxicity on MG-63 observed by 24 hrs extract of 2Seal which decreased in favor of AH Plus with 72 hrs extract (Fig. 2). Results obtained by trypan blue assays showed similar pattern of decrease in cell numbers (Fig. 3, 4) 

## Discussion

Evaluation of cytotoxity induced by root canal filling materials using human cell lines has been widely used in recent decades to predict and compare the new materials biocompatibilities (4, 6). In the current study we compared cytotoxicity of two common used root canal sealers (AH Plus and 2Seal) on 2 fibroblast like cell lines (Saos-2 and MG-63). Based on previous investigations conducted worldwide, AH Plus had showed prior biocompatibility to many other resin based sealers (11, 17, 22, 23). Our results show that both sealers with 24 or 72 hrs h extraction times have the same cytotoxic effects on Saos-2 cell line, however on MG-63 cell line, 2Seal shows more cytotoxicity with 24 hrs extract while AH Plus is more toxic after 72 h extraction. 

Despite of worldwide AH Plus’ common use, it causes cytotoxic effects on different cell lines, like other root canal filling materials (e.g. on murine fibroblast, V79, Hela...) (8, 10, 17, 18, 24, 25). The in vitro method chosen to test the cytotoxicity of root filling materials could significantly influence the obtained final results. Freshly made mixtures usually show more cytotoxic properties than their set forms (11, 18, 24). These mixtures might disintegrate in the liquid media used in the cell culture and do not necessarily resemble the normal in vivo condition existing in human dental canal. Using set discs of AH Plus and 2Seal with same size and shape keeps the extraction ratio of surface area per extraction volume constant, hence elute concentration would be only dependent on the nature of the dissolved substances. 

It has been shown that with different sealer materials, extraction time might have great effects on the final observed cytotoxicities. For instance with 24 hrs extraction time, Endion has showed higher toxicity on Saos-2 cell line compared to AH Plus, while with 72 hrs extraction time, AH Plus toxicity exceeded (26). AH Plus is an epoxy resin-based sealer which seems to release more toxic substances into the medium during 72 hrs incubation, hence its cytotoxicty increases by time compared to a silicone-based sealer (e.g. Roeko Seal Automix) (17). We found similar results with MG-63 cell line, where AH Plus showed higher cytotoxicity with 72 hrs extract compared to 2Seal that showed higher toxicity with 24 hrs extract (Fig. 2, 4). However, such a difference was not statistically significant in Saos-2 cell line which might be due to the difference in sensitivity of these two cell lines to the eluted toxic compounds (12) and/or the instability of eluted toxic substances in the media. The instability could be due to the fact that formaldehyde and other volatile species might leave the media by warm incubation (22). 

Alternatively some reactive species might cross react with the serum proteins in the media with consequent decrease in toxicity during extraction time. Since AH Plus setting process is the result of a polymerization system called “Linear Epoxide-Amine Addition”, leaching non reacted monomers from this polymer matrix, might be another justification for its constant increasing (however less than the other sealing materials) cytotoxicity with longer extraction time (4, 27, 28). 

On the other hand, there is little information concerning 2Seal cytotoxicity on different cell lines especially Saos-2 and MG-63 cell lines. The information provided by the manufacturer indicates that 2Seal (like AH Plus) is a polymer made from interaction between a bisphenol derivative with a diamine derivative. It is an epoxy resin sealer and leftover of both these two copolymers are toxic and might be responsible for 2Seal cytotoxicity. In an in vivo study conducted with 3 different sealers on rat molars, 2Seal showed the best biocompatibility in terms of producing less periapical inflammation compared to the other two sealers: RSA Roekoseal and Aptal Harz (29). 

In another study, 2Seal had showed histological effects similar to AH Plus on canine molars periapical tissues (23). In the present study, at least on Saos-2 cell line, both sealers showed similar toxicities. Different sensitivities to sealing materials elutes observed in different cell lines has been reported in many other studies (12, 21, 30) and it has been recommended to perform cytotoxicity studies on different cell lines before any discrete judgment about biocompatibility of different biomaterials (12).The difference between kinetic of toxic substances release from solidified polymers might be the reason for slight differences observed by different extraction times with MG-63 cell line. However, the difference between each cell line susceptibility to the type of elutes should not be ignored as well.

## Notes

Authors declare no conflict of interest.

## References

[1] Grossman LI (1976). Endodontics 1776-1976: a bicentennial history against the background of general dentistry. J Am Dent Assoc.

[2] Beltes P, Koulaouzidou E, Kolokuris I (1997). In vitro evaluation of the cytotoxicity of two glass-ionomer root canal sealers. J Endodont.

[3] Cohen SH (2006). Pathway of the Pulp.

[4] Goldberg M (2008). In vitro and in vivo studies on the toxicity of dental resin components: a review. Clinical Oral Investigations.

[5] Hauman CH, Love RM (2003). Biocompatibility of dental materials used in contemporary endodontic therapy: a review. Part 2. Root-canal-filling materials. Int Endod J.

[6] Modena KC, Casas-Apayco LC, Atta MT (2009). Cytotoxicity and biocompatibility of direct and indirect pulp capping materials. J Appl Oral Sci.

[7] Gambarini G, Testarelli L, Al-Sudani D (2011). In vitro Evaluation of the Cytotoxicity of Different Root Canal Filling Materials. Open Dent J.

[8] Willershausen I, Callaway A, Briseno B (2011). In vitro analysis of the cytotoxicity and the antimicrobial effect of four endodontic sealers. Head & Face Medicine.

[9] Huang FM, Tai KW, Chou MY (2002). Cytotoxicity of resin-, zinc oxide-eugenol-, and calcium hydroxide-based root canal sealers on human periodontal ligament cells and permanent V79 cells. Int Endod J.

[10] Spangberg L, Langeland K (1973). Biologic effects of dental materials. 1. Toxicity of root canal filling materials on HeLa cells in vitro. Oral Surg Oral Med Oral Pathol.

[11] Miletic I, Jukic S, Anic I (2003). Examination of cytotoxicity and mutagenicity of AH26 and AH Plus sealers. Int Endod J.

[12] Pissiotis E, Spangberg LS (1991). Toxicity of Pulpispad using four different cell types. Int Endod J.

[13] Freshney RI, Freshney RI (2005). Culture of animal cells: a manual of basic technique. Cytotoxicity.

[14] Rodan SB, Imai Y, Thiede MA (1987). Characterization of a human osteosarcoma cell line (Saos-2) with osteoblastic properties. Cancer Res.

[15] Pautke C, Schieker M, Tischer T (2004). Characterization of osteosarcoma cell lines MG-63, Saos-2 and U-2OS in comparison to human osteoblasts. Anticancer Res.

[16] Granchi D, Stea S, Ciapetti G (1995). Endodontic Cements Induce Alterations in the Cell-Cycle of in-Vitro Cultured Osteoblasts. Oral Surg Oral Med O.

[17] Oztan MD, Yilmaz S, Kalayci A (2003). A comparison of the in vitro cytotoxicity of two root canal sealers. J Oral Rehabil.

[18] Lodiene G, Morisbak E, Bruzell E (2008). Toxicity evaluation of root canal sealers in vitro. Int Endod J.

[19] Roydent Dental Products (2003).

[20] Mosmann T (1983). Rapid colorimetric assay for cellular growth and survival: application to proliferation and cytotoxicity assays. J Immunol Methods.

[21] Cenni E, Ciapetti G, Granchi D (1999). Established cell lines and primary cultures in testing medical devices in vitro. Toxicol In Vitro.

[22] Oysaed H, Ruyter IE, Sjovik Kleven IJ (1988). Release of formaldehyde from dental composites. J Dent Res.

[23] Leonardo MR, da Silva LAB, Almeida WA (1999). Tissue response to an epoxy resin-based root canal sealer. Endod Dent Traumatol.

[24] Zoufan K, Jiang J, Komabayashi T (2011). Cytotoxicity evaluation of Gutta Flow and Endo Sequence BC sealers. Oral Surgery Oral Medicine Oral Pathology Oral Radiology and Endodontology.

[25] Merdad K, Pascon AE, Kulkarni G (2007). Short-term cytotoxicity assessment of components of the epiphany resin-percha obturating system by indirect and direct contact millipore filter assays. J Endodont.

[26] Torres JL, Alvardo M, Herrera P, VALLEJO, VILLENA H, SARAVIA-CUNZA M (2003). The Cytotoxicity Evaluation of Five Root Canal Sealers in Saos-2 Cells.

[27] Klee JH, Salomone JC RB Linear Epoxide-Amine Addition Polymers. Polymeric Materials Encyclopaedia1996.

[28] Brackett MG, Marshall A, Lockwood PE (2008). Cytotoxicity of endodontic materials over 6-weeks ex vivo. Int Endod J.

[29] Dammaschke T, Schneider U, Stratmann U (2006). Reaction of inflamed periapical tissue to three different root canal sealers. Deutsche Zahnärztliche Zeitschrift.

[30] Thonemann B, Schmalz G, Hiller KA (2002). Responses of L929 mouse fibroblasts, primary and immortalized bovine dental papilla-derived cell lines to dental resin components. Dent Mater.

